# Cold Responsive Gene Expression Profiling of Sugarcane and *Saccharum spontaneum* with Functional Analysis of a Cold Inducible *Saccharum* Homolog of NOD26-Like Intrinsic Protein to Salt and Water Stress

**DOI:** 10.1371/journal.pone.0125810

**Published:** 2015-05-04

**Authors:** Jong-Won Park, Thiago R. Benatti, Thiago Marconi, Qingyi Yu, Nora Solis-Gracia, Victoria Mora, Jorge A. da Silva

**Affiliations:** 1 Texas A&M AgriLife Research, The Texas A&M System, Weslaco, Texas, United States of America; 2 Texas A&M AgriLife Research, The Texas A&M System, Dallas, Texas, United States of America; Institute of Genetics and Developmental Biology, Chinese Academy of Sciences, CHINA

## Abstract

Transcriptome analysis of sugarcane hybrid CP72-1210 (cold susceptible) and *Saccharum spontaneum* TUS05-05 (cold tolerant) using Sugarcane Assembled Sequences (SAS) from SUCEST-FUN Database showed that a total of 35,340 and 34,698 SAS genes, respectively, were expressed before and after chilling stress. The analysis revealed that more than 600 genes are differentially expressed in each genotype after chilling stress. Blast2Go annotation revealed that the major difference in gene expression profiles between CP72-1210 and TUS05-05 after chilling stress are present in the genes related to the transmembrane transporter activity. To further investigate the relevance of transmembrane transporter activity against abiotic stress tolerance, a *S*. *spontaneum* homolog of a NOD26-like major intrinsic protein gene (*SspNIP2*) was selected for functional analysis, of which expression was induced after chilling stress in the cold tolerant TUS05-05. Quantitative real-time PCR showed that *SspNIP2* expression was increased ~2.5 fold at 30 minutes after cold treatment and stayed induced throughout the 24 hours of cold treatment. The amino acid sequence analysis of the cloned *SspNIP2* confirmed the presence of six transmembrane domains and two NPA (Asn-Pro-Ala) motifs, signature features of major intrinsic protein families. Amino acid analysis confirmed that four amino acids, comprising the ar/R (aromatic residue/arginine) region responsible for the substrate specificity among MIPs, are conserved among monocot silicon transporters and SspNIP2. Salinity stress test on SspNIP2 transgenic tobacco plants resulted in more vigorous transgenic lines than the non-transgenic tobacco plants, suggesting some degree of tolerance to salt stress conferred by SspNIP2. SspNIP2-transgenic plants, exposed to 2 weeks of water stress without irrigation, developed various degrees of water stress symptom. The water stress test confirmed that the SspNIP2 transgenic lines had lower evapotranspiration rates than non-transgenic lines, suggesting that SspNIP2 transgenic lines showed a slight tolerance to the early water stress compared to wild type plants.

## Introduction

Sugarcane (*Saccharum* spp. L.) is a perennial grass that is widely cultivated in tropical and subtropical regions around the world as a major source for raw sugar. It is estimated that approximately 70% of the global sugar production is derived from sugarcane, while the remaining is derived from sugar beet cultivated in temperate regions [1]. Combining favorable traits from wild species, such as *S*. *spontaneum*, into a common sugarcane genotype is certainly desirable to expand the cultivation area, which is restricted to subtropical and tropical regions, despite its tremendous yield potential. This process would improve its cold tolerance, resulting in less energy input, with no genetic modification. In the main land U.S., for instance, the production of sugar from sugarcane is restricted to the states of Florida, Louisiana, and Texas. To expand the acreage beyond the southern half of the U.S., cold tolerance is crucial. Modern commercial sugarcane cultivars (2n = 100–130) are derived from the interspecific hybridization between *S*. *officinarum*, which is high in sucrose, and *S*. *spontaneum*, tolerant to a broad range of biotic and abiotic stresses, followed by a series of backcrossing with other *S*. *officinarum* accessions [2–4]. Although the interspecific hybridization significantly contributed to establish modern sugarcane cultivars, the high degree of polyploidy and the narrow gene pool of modern cultivars imposed difficulties on the effort of sugarcane breeders to develop new sugarcane cultivars with higher yield or enhanced disease resistance.

In addition to being a source of raw sugar, sugarcane has long been used for ethanol production in Brazil by the fermentation of its juice [5, 6]. Due to the limited amount of fossil fuel left in nature, there is an increasing consensus among scientists and government officials to incorporate lignocellulosic biomass as a feedstock for biofuel production. At present, the global sugarcane cultivation provides the largest scale of lignocellulosic biomass than any other crop species including potential bioenergy feedstock crops, such as *Miscanthus* and switchgrass [1, 6, 7]. Despite all the superior traits of sugarcane as a lignocellulosic biomass feedstock crop, adopting commercial sugarcane hybrids as a dedicated biomass crop for biofuel production is limited only to certain parts of the world due to its lack of cold and drought stress tolerance.

Currently, many sugarcane breeding programs around the world are trying to incorporate more diverse germplasms, including sorghum and other closely related wild grass species, belonging to the *Saccharum* complex [8–10], into the genetic background of modern sugarcane cultivars in an attempt to increase the genetic diversity [8, 11, 12]. The *Saccharum* complex includes the genus *Saccharum* and four closely related genera such as *Erianthus* sect. *Ripidium* (2n = 20–60), *Sclerostachya* (2n = 30), *Narenga* (2n = 30) and *Miscanthus* sect. *Diandra* (2n = 38–76) [9, 10, 13, 14], which are amenable to be crossed with *Saccharum* species. Although the *Saccharum* complex is considered as a valuable resource for genetic improvement of commercial sugarcane hybrids, only limited number of success has been reported [14].

*S*. *spontaneum* is a highly adaptable species showing diverse phenotypes that can grow in diverse habitats such as drought, cold and high salt environments [15], illustrating its wide range of genetic variability. As shown in the interspecific hybridization taken place in the late 19th century, leading to the development of modern sugarcane hybrids, the incorporation of genetic traits from *S*. *spontaneum* into sugarcane hybrids appears to be less problematic compared to other genera within the *Saccharum* complex. Wild populations of *S*. *spontaneum*, the species with a very wide geographic distribution, present the greatest potential source of genetic variation and have played an important role in the development of modern sugarcane cultivars [4]. However, a limited number of *S*. *spontaneum* genotypes has been used in the production of modern sugarcane cultivars [4]. Only two genotypes of *S*. *spontaneum* were used in the initial crosses made in the late 19th century and early 20th century in India and Java [16], resulting in a narrow genetic basis for modern sugarcane varieties, as it has been demonstrated with molecular markers by various authors [17–19].

The current study exploited the diverse genetic variability of *S*. *spontaneum* in order to fabricate a better strategy specifically targeting the improvement of abiotic stress tolerance in sugarcane hybrids. This was done through the investigation of gene expression profiles, by RNA-Seq, of a cold tolerant genotype of *S*. *spontaneum*, TUS05-05, and a cold susceptible sugarcane cultivar, CP72-1210. The study compared the gene expression profiles of each genotype before and after chilling stress and investigated genes responsive to cold stress, followed by a functional analysis of a selected gene in transgenic tobacco plants. The data showed that more than six hundred different genes were differentially expressed in each genotype after chilling stress. Blast2Go analysis showed that the major difference in gene expression profiles between CP72-1210 and TUS05-05 was in the genes involved in transmembrane transporter activity. Among those differentially expressed genes, we selected a *Saccharum* homolog of *NOD26-like intrinsic protein 2* (*SspNIP2*), which was induced after chilling stress in the cold tolerant genotype, TUS05-05, for functional analysis in a heterologous plant system. The amino acid sequence and phylogenetic analyses indicated that SspNIP2 has a homology with monocot silicon transporters. We investigated the function of SspNIP2 in transgenic tobacco plants under salt and water stress conditions as it has been shown that silicon application improves a broad range of biotic and abiotic stress tolerance in plants [20].

## Materials and Methods

### Chilling stress treatment, tissue harvest and RNA-Seq

Sugarcane cultivar CP72-1210 and *S*. *spontaneum* TUS05-05 were germinated from lateral buds and maintained in 2 gallon pots in the greenhouse. Plants that were 8 weeks old were transferred to a growth chamber under 16 hours (hrs) of light per day at 30°C/28°C (day/night) and were kept in the growth chamber for 3 days before exposing them to chilling stress for 20 hours at 1.5°C [21]. Leaf tissue samples were collected before and after chilling stress for RNA extraction using RNeasy Plant Mini kit (Qiagen). The total RNAs were treated with DNase I (Qiagen) to remove any DNA from RNA samples. The cDNA sequencing library preparation [22] and single-end 100 base pair (bp) were conducted at the Texas A&M AgriLife Genomics and Bioinformatics Institute, using Illumina GAIIx following the manufacturer's instruction (Illumina Inc.).

### RNA-Seq data analysis and Blast2Go annotation

The RNA-Seq raw data was processed by CLC Bio Genomics Workbench following the manufacturer's instruction. Briefly, the sequencing reads were mapped to the Sugarcane Assembled Sequences (SAS) from SUCEST-FUN Database (http://sucest-fun.org) as a reference (mapping parameters: minimum length fraction = 0.7, minimum similarity fraction = 0.8, maximum number of hits for a read = 1). The gene expression value of each mapped SAS was determined by RPKM (reads per kilobase of exon model per million reads mapped). The gene expression values of each SAS in each genotype before and after chilling stress were compared and statistically evaluated using a Z-test. A list of differentially expressed genes (DEs) was prepared based on the fold change greater than ±2 after the stress, whose adjusted p-value of false discovery rate (FDR) was less than 0.001. To validate RNA-Seq data, quantitative real-time PCR (qRT-PCR) was conducted using six SAS sequences (SCRLSD2009F04.g; SCACLB1046H03.g; SCCCCL3080G01.g; SCEPLB1042E05.g; SCRFRZ3058E03.b; SCUTSD1028A10.g). The primer sequences are listed on S1 Fig legend. For qRT-PCR, five TUS05-05 plants were exposed to chilling stress at 0°C for 20 hours. The total RNA was extracted with Qiagen RNeasy Plant minikit from leaves harvested before and after chilling treatment following the manufacturer's instruction. The RNA samples were pooled for the first strand synthesis followed by PCR (BioRad iScript RT supermix and SYBR supermix).

The SAS sequences that passed the DE criteria were functionally annotated using Blast2Go with default settings. The DE SAS sequences were blasted against the GenBank database, followed by mapping to the GO (Gene Ontology) information of blast hits. Blast2Go functional annotation was analyzed by generating Blast2Go combined graphs of molecular function and biological process with default settings. KEGG (Kyoto Encyclopedia of Genes and Genomes) pathway mapping was also conducted by KEGG mapping tool in Blast2Go with default settings.

### Cloning of *SspNIP2* and amino acid sequence analysis

Full length *SspNIP2* was obtained by 5' and 3' Rapid Amplification of cDNA Ends (RACE; Clontech SMARTer RACE kit). The cloned SspNIP2 was blasted to GenBank database to confirm its identity. The amino acid sequence analysis of SspNIP2 was conducted by MPEx (Membrane Protein Explorer version 3.2.11). ClustalW2 was used for the amino acid sequence alignment between SspNIP2 and monocot silicon transporters, ZmLsi6 (NM001111550), HvLsi6 (AB447484.1),OsLsi6 (AB253627.1) and OsLsi1 (AB222272.1). Phylogenetic analysis of SspNIP2 with monocot silicon transporters, ZmLsi6, HvLsi6, OsLsi6, OsLsi1, ZmLsi1 (DQ524811.1), TaLsi1 (HM803114.1), and HvLsi1 (AB447482.1) was conducted by Maximum Likelihood Method using MEGA6 [23].

### Chilling stress and qRT-PCR for the analysis of *S*. *spontaneum* homolog of NIP2 expression

To validate the RNA-Seq data, a set of five TUS05-05 plants was exposed to 0°C, the leaf tissue samples were collected at 0 hr, 0.5 hr, 1 hr, 4 hr, 9 hr and 24 hr after the chilling stress. In addition, plants were placed under normal growing conditions after the chilling stress, from which tissue samples were collected at 24 hrs after returning to the normal growth condition. The total RNAs were extracted using Qiagen RNeasy Plant Mini kit and treated with DNase I. The RNA samples prepared from five plants were pooled and used for reverse transcription (BioRad iScript RT supermix), then quantitative real-time PCR (qRT-PCR) was conducted to investigate the gene expression dynamics of *SspNIP2* under chilling stress using a primer set, NIP2/F (5'-gtagaacggcacctgaatcc-3') and NIP2/R (5'-gaggacaacaagcgcatctc-3').

### Generation of transgenic tobacco plants

The *SspNIP2* gene was cloned in between dual 35S promoter and 35S terminator in pBIN(+) background (BIN/SspNIP2). *Agrobacterium tumefaciens* EHA105 was transformed with BIN/SspNIP2 by electroporation and used for transformation of *Nicotiana tabacum* plant. The fully expanded young leaves of 4–8 weeks old tobacco plants, grown in a magenta box on MS medium, were collected for leaf disc (~5 mm in diameter) preparation in MS medium, supplemented with 15 g/l of sucrose (MS-15). The overnight grown *Agrobacterium* EHA105 in LB medium was harvested by centrifugation at 5000 rpm for 20 min and resuspended in MS-15 to OD 0.4–0.6 at 595nm. The tobacco leaf discs were transferred into the *Agrobacterium* suspension and incubated for ~10 minutes with gentle shaking. The leaf discs were briefly blot dried on autoclaved Whatman filter paper and placed on MS solid medium supplemented with 30 g/l sucrose, 1 mg Benzyl amino purine (BAP), 1 mg of Naphthalenic acid (NAA) and 2.5 g/l gelrite for co-cultivation for 3 days in a growth chamber. Then, the leaf discs were transferred onto a regeneration medium (MS supplemented with 30 g/l sucrose, 2 mg BAP, 1 mg NAA, 100 mg Kanamycin, and 500 mg Cefotaxime in 1 L of MS solid medium). The explants were subcultured on the regeneration medium every 2 weeks. When regenerated shoots appeared on the explants, the shoots were cut and transferred to the selective medium (MS medium supplemented with 30 g/l sucrose, 100 mg Kanamycin, 500 mg Cefotaxime and 500 mg Timentin per 1 L medium) for root regeneration. The regenerated shoots were subcultured on the regeneration medium until roots regenerated. The regenerated plants were transferred to soil, and transformants were screened by PCR and qRT-PCR.

### Salt and water stress condition

The T3 SspNIP2 transgenic tobacco and non-transgenic plants were germinated and grew in a pot filled with 80 g of soil. For salt stress, two to three plants of each six-week old transgenic (NIP2 #6, #7, #8 and #9) and non-transgenic lines were irrigated with 100 ml of 200 mM NaCl for 4 weeks, followed by 100 ml of 250 mM NaCl for 2 weeks. For water stress experiment, six-week old transgenic lines, 6 plants of NIP2 #6, 5 plants of NIP2 #7, 2 plants of NIP2 #8, and 3 plants of NIP2 #9, and 3 non-transgenic tobacco plants were subjected to water stress for 2 weeks without irrigation, and the moisture content retained in individual pots were monitored everyday by measuring the weight of each pot. The comparison of relative moisture content of transgenic and nontransgenic lines subjected to water stress was conducted and statistically evaluated by F-test followed by t-test.

## Results

### Transcriptome analysis of sugarcane cultivar CP72-1210 and *S*. *spontaneum* TUS05-05 after chilling stress

**Fig 1:**
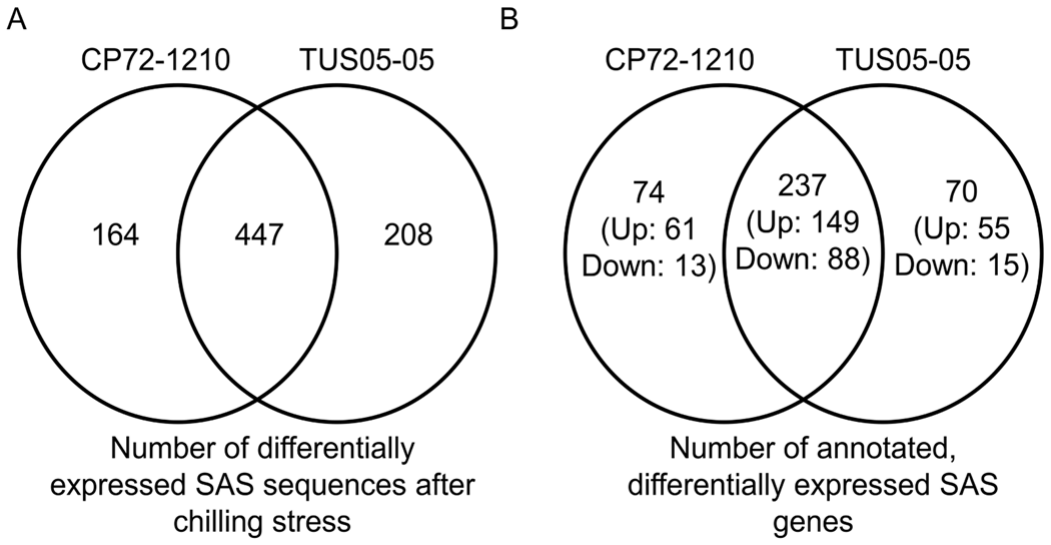
Venn diagram of differentially expressed genes in CP72-1210 and TUS05-05. **A.** Venn diagram of differentially expressed SAS sequences before gene annotation. **B.** Venn diagram of annotated genes differentially expressed in each genotype. The number of uniquely and commonly expressed genes in each genotype is indicated in numbers in the diagram. Up and Down indicate up-regulation and down-regulation, respectively.

We compared the transcriptomes of a cold susceptible sugarcane CP72-1210 and a cold tolerant *S*. *spontaneum* TUS05-05, before and after chilling treatment at 1.5°C for 20 hours. A total of 179.8 million reads were obtained by quality filtering, and of those, 88.4 million reads (49.2%) were mapped to the sugarcane assembled sequences (SAS) from SUCEST-FUN Database (http://sucest-fun.org) (Table 1). The RNA-Seq analysis revealed that each treatment generated a list of 35,340 and 34,698 SAS sequences, expressed before and after chilling stress in CP72-1210 and TUS05-05, respectively, which were further analyzed to determine the SAS sequences that were differentially expressed between treatments (Table 2). The analysis showed that ~80% of the 43,141 SAS sequences were expressed before and after chilling treatment in each genotype (Table 2). The number of differentially expressed SAS sequences showing fold change greater than ±2 after chilling stress was 611 in CP72-1210 and 655 in TUS05-05 (Table 2). The analysis confirmed that 447 SAS sequences were differentially expressed commonly in both CP72-1210 (73%) and TUS05-05 (68%) (Fig 1A). To validate the transcriptome data, quantitative real-time PCR (qRT-PCR) was conducted with six SAS sequences that were up-regulated in TUS05-05 (S1 Fig). The relative expression levels of four of six SAS sequences in qRT-PCR were ranged from 37% to 72% of their fold increases in RNA-Seq analysis (S1 Fig), and the other two qRT-PCR data were less than 20% of those obtained from RNA-Seq analysis (S1 Fig). Although variation was observed in the relative gene expression levels obtained from qRT-PCR and RNA-Seq, the up-regulation of six SAS sequences was confirmed by qRT-PCR (S1 Fig). However, these results also indicated that the validation of RNA-Seq data by qRT-PCR should be performed before genes of interest are further investigated.

**Table 1:** Summary of RNA-Seq data of sugarcane CP72-1210 and *S*. *spontaneum* TUS05-05.

	CP72-1210 before chilling	CP72-1210 after chilling	TUS05-05 before chilling	TUS05-05 after chilling
Total number of reads (M^a^) after quality filtering	29.9	56.0	32.1	61.8
Total number of reads (M) mapped to SAS^b^ (%)	15.5 (51.8%)	29.4 (52.5%)	14.9 (46.5%)	28.6 (46.3%)
Total number of reads (M) unmatched to SAS (%)	14.4 (48.2%)	26.6 (47.5%)	17.2 (53.5%)	33.2 (53.7%)

^a^M = Million reads

^b^SAS = Sugarcane Assembled Sequences from SUCEST-FUN Database (http://sucest-fun.org) at Instituto de Química—Universidade de São Paulo, funded by FAPESP Bioenergy Research Program BIOEN (http://bioenfapesp.org).

**Table 2:** Summary of differential gene expression analysis.

	Comparison across treatment	
	CP72-1210 before vs. after chilling	TUS05-05 before vs. after chilling
Total number of SAS genes	43,141	
Number of SAS genes expressed (%)	35,340 (81.9%)	34,698 (80.4%)
Number of differentially expressed (DE*) SAS genes	611	655

*DE criteria: fold change ≥ ±2 after chilling treatment, FDR (false discovery rate) < 0.001.

### Functional annotation of differentially expressed genes

**Fig 2:**
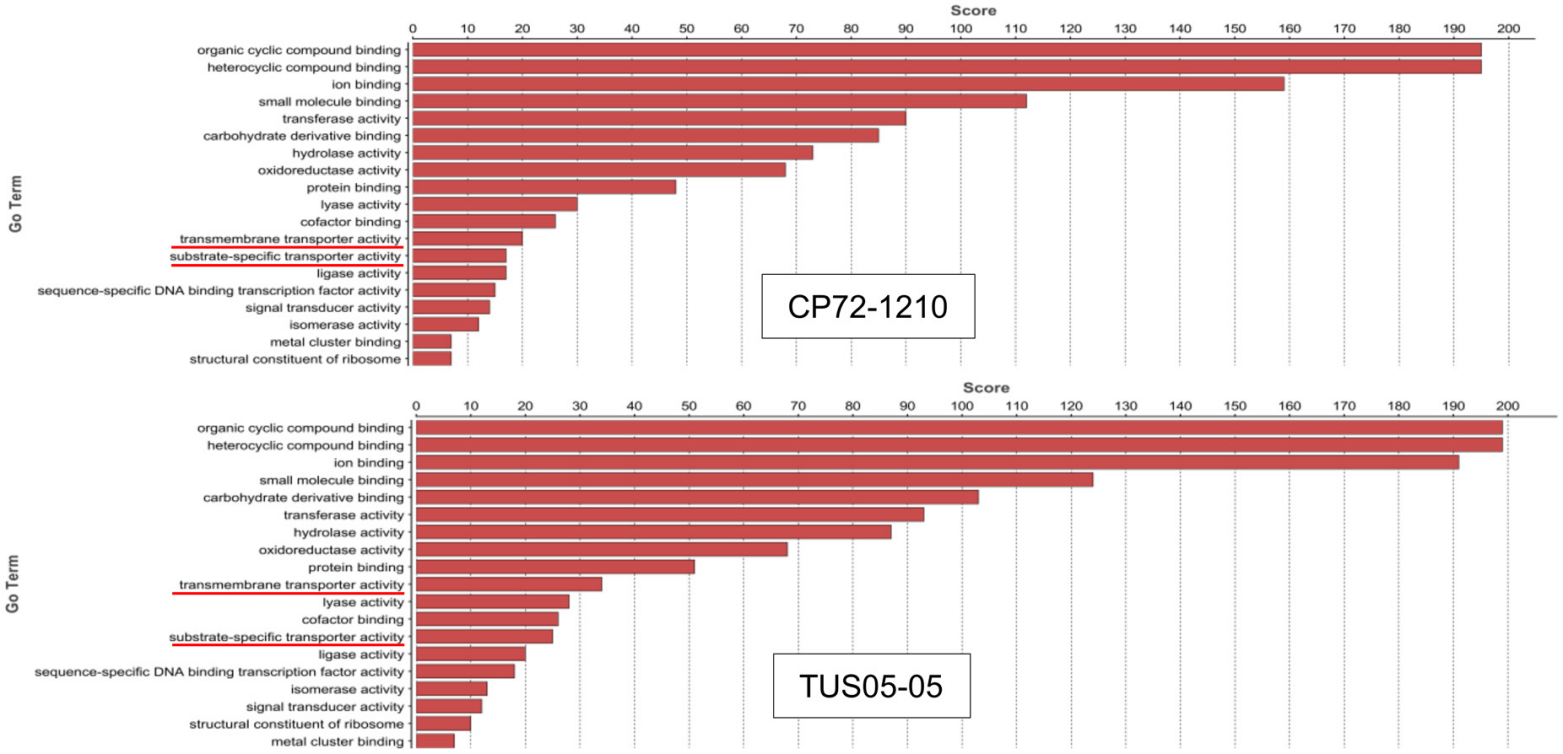
Analysis of gene annotation of differentially expressed genes after chilling stress. The annotated sequences of CP72-1210 (top) and TUS05-05 (bottom) were analyzed based on the molecular function of GO terms by Blast2Go. GO terms are listed on the left, and the Blast2Go score of molecular function at level 3 is shown on top. The molecular function of transmembrane transporter activities are underlined in red.

The functional annotation by Blast2Go confirmed that 311 (~51%) of 611 SAS sequences differentially expressed in CP72-1210 and 307 (~47%) of 655 SAS sequences in TUS05-05 were successfully annotated (Fig 1B and S1 Table). The Blast2Go analysis also showed that the most common molecular functions among those differentially expressed genes, 311 in CP72-1210 and 307 in TUS05-05, were related to either binding (44% in TUS05-05 and 46% in CP72-1210) or catalytic activities (39% in both TUS05-05 and CP72-1210) (data not shown). KEGG pathway mapping indicated that in both genotypes, the metabolic pathways involved in starch and sucrose metabolism, purine and phenylalanine metabolism and phenylpropanoid biosynthesis were the most affected metabolic pathways by chilling stress (S2 Table). According to the gene annotation analysis based on molecular function by Blast2Go, the most noticeable differences between CP72-1210 and TUS05-05 in the molecular function of those differentially expressed genes were in the genes related to the transporter activities (Fig 2). Among those annotated genes in CP72-1210 and TUS05-05, 237 genes were differentially expressed common in both genotypes (Fig 1B), of which 149 and 88 genes were up- and down-regulated after chilling stress, respectively (Fig 1B and S1 Table). The functional annotation of these 237 genes showed that the overall annotation pattern based on molecular function is similar to the ones obtained with differentially expressed 311 genes in CP72-1210 and 307 genes in TUS05-05 in Fig 2, except transporter activities (S2 Fig). This data suggested that the difference in molecular function of transporter activities between CP72-1210 and TUS05-05 is likely due to the genes differentially expressed in either CP72-1210 or TUS05-05.

### Analysis of differentially expressed genes unique in CP72-1210 and TUS05-05

The transcriptome analysis revealed that 74 of 311 annotated genes in CP72-1210 and 70 of 307 genes in TUS05-05 were uniquely differentially expressed in each genotype (Fig 1B and S1 Table). Of those genes, 13 (17.6%) of 74 genes in CP72-1210 and 15 (21.4%) of 70 genes in TUS05-05 were down-regulated after chilling stress (Fig 1B and S1 Table). The gene annotation analysis of these down-regulated genes based on biological process indicated that in CP72-1210, the biological process for stress response was greatly compromised unlike TUS05-05 whose metabolic pathway was the most affected by chilling stress (S3 Fig). This annotation data based on biological process category is consistent with the data that various genes related to the stress response (ethylene, salt and cold) were down-regulated in the cold-sensitive CP72-1210 (S1 Table).

Gene annotation analysis of those genes up-regulated in CP72-1210 (61 of 74 genes) and TUS05-05 (55 of 70 genes) after chilling stress showed that the cold tolerant TUS05-05 had an increased level of transporter activity compared to the cold sensitive CP72-1210 (S4 Fig). This indicated that the increased transporter activity in TUS05-05 was due to uniquely up-regulated genes for transporter activities in TUS05-05. S1 Table showed a list of genes of which products could be associated with cellular membranes that may function as membrane-bound transporters. The expression level of those genes highlighted in S1 Table changed ≥ 2 fold after chilling stress in TUS05-05 (FDR ≤ 0.001) while the change in CP72-1210 was insignificant (data not shown). Among those genes highlighted in S1 Table, we selected a *S*. *spontaneum* homolog of NOD26-like intrinsic protein gene (*SspNIP2*), a member of aquaporin gene family [24], to investigate its function under various abiotic stress conditions in plant. Since NIP2 mediates intercellular trafficking of water and other small solutes, SspNIP2 may play a role on plant response toward osmotic pressure changes in plants caused by various abiotic stress conditions such as water and salt stress.

### A *S*. *spontaneum* homolog of NIP2 (*SspNIP2*) is similar to monocot silicon transporters and induced by cold treatment

**Fig 3:**
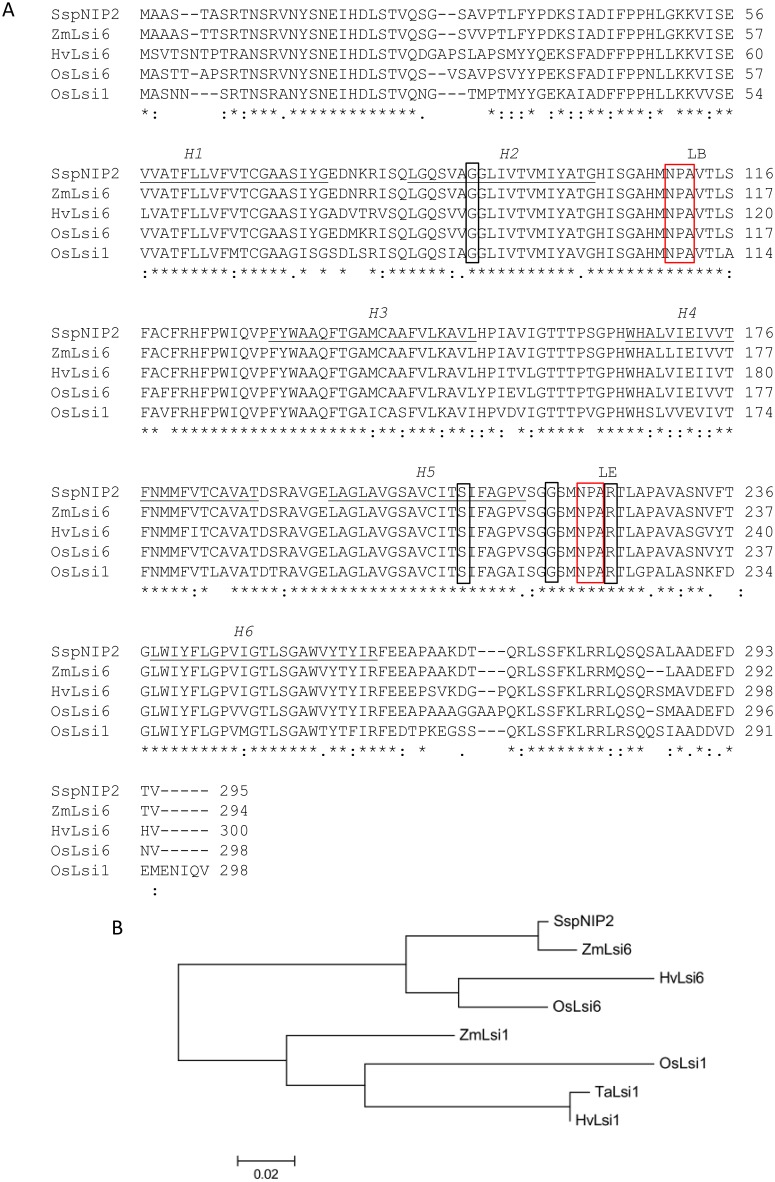
SspNIP2 amino acid sequence analysis. **A.** Amino acid sequence alignment of SspNIP2 with monocot silicon transporters; Maize ZmLsi6 (NM001111550), Barley HvLsi6 (AB447484.1), Rice OsLsi6 (AB253627.1) and OsLsi1 (AB222272.1). Six transmembrane domains, H1 to H6, were predicted by Membrane Protein Explorer version 3.2.11. Two NPA motifs and four amino acid residues constituting ar/R selectivity filter of SspNIP2 and monocot silicon transporters are indicated in red and black open boxes, respectively. **B.** Phylogenic analysis of SspNIP2 with monocot silicon transporters; Maize ZmLsi6 (NM001111550) and ZmLsi1 (DQ524811.1), Barley HvLsi6 (AB447484.1) and HvLsi1 (AB447482.1), Rice OsLsi6 (AB253627.1) and OsLsi1 (AB222272.1), and Wheat TaLsi1 (HM803114.1). Phylogenetic tree was generated by Maximum Likelihood method using MEGA6 [23].

The full-length sequence of an 888 nucleotide-long *SspNIP2* was obtained by rapid amplification of cDNA ends. The amino acid sequence analysis confirmed the presence of six transmembrane domains (Helix (H) 1 to H6) and two NPA (Asn-Pro-Ala) motifs in loop B and loop E, each connecting H2 to H3, and H5 to H6, respectively, which are main key features of major intrinsic protein gene families (MIPs) (Fig 3A). The BlastP search with SspNIP2 amino acid sequence showed that SspNIP2 has 98% identity with *Zea mays* NIP2;2 (ZmNIP2;2) that is also known as a silicon transporter, ZmLsi6, localized in the xylem parenchyma cells in Maize leaf tissue [25, 26]. Amino acid sequence alignment of SspNIP2 with monocot silicon transporters, ZmLsi6, *Hordeum vulgare* Lsi6 (HvLsi6), *Oryza sativa* Lsi6 (OsLsi6) and Lsi1 (OsLsi1) showed that their amino acid sequences are highly conserved (Fig 3A). The sequence alignment also revealed that the four amino acid residues (G-S-G-R) of the ar/R (aromatic residue/arginine) region that governs the substrate specificity of MIPs [27] are conserved among those monocot silicon transporters and SspNIP2 (Fig 3A). Molecular phylogenetic analysis of SspNIP2 with monocot silicon transporters showed that SspNIP2 is grouped with monocot Lsi6 (Fig 3B), a silicon transporter responsible for silicon unloading from xylem vessel in leaf tissue [26]. These data suggested that SspNIP2 may have a function similar to those of monocot silicon transporters in *S*. *spontaneum*, but further analysis is needed to identify the substrate of *SspNIP2*.

**Fig 4:**
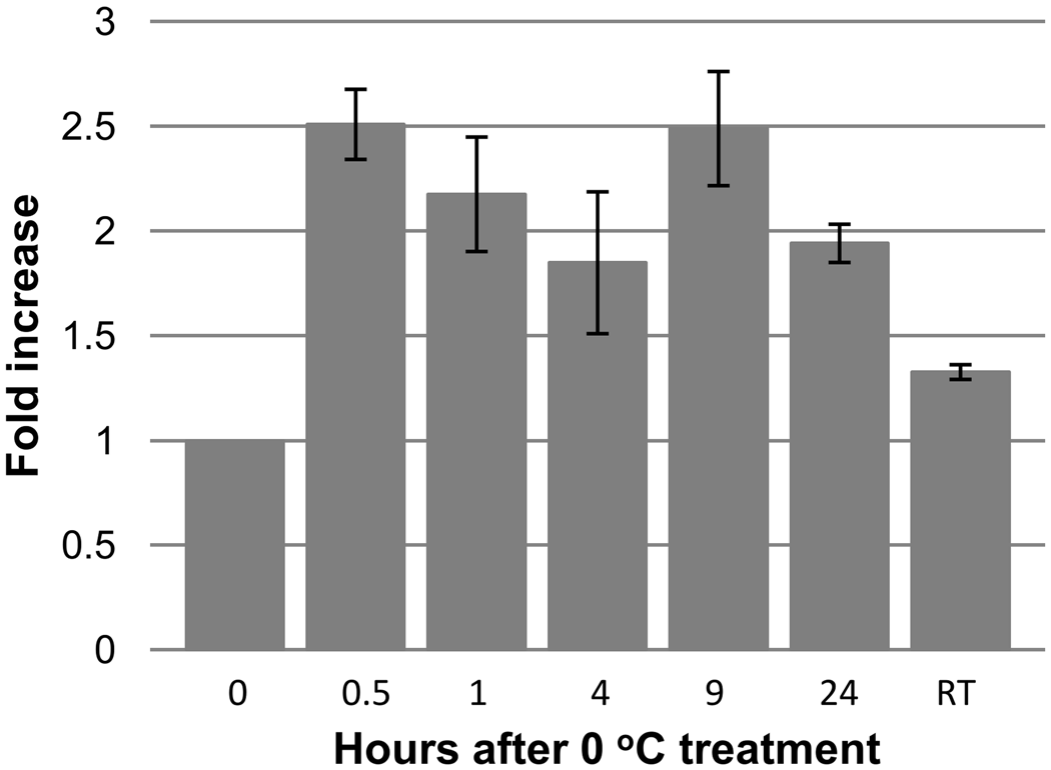
Expression profile of *SspNIP2* in *S*. *spontaneum* TUS05-05 during 24 hrs of cold treatment. Time course experiment was conducted by qRT-PCR to study the dynamics of SspNIP2 gene expression profile during cold treatment. Five TUS05-05 plants were subjected to cold treatment at 0°C, and leaf tissue samples were collected at different time point as shown on the Fig for RNA preparation. RNA samples from five individual plants were pooled for qRT-PCR. qRT-PCR was conducted three times with triplicates each time. RT indicated the RNA sample prepared from plants that were returned to normal growth condition after cold treatment.

In order to investigate the gene expression dynamics of *SspNIP2* under chilling stress, a set of five TUS05-05 plants were exposed to 0°C, from which leaf tissue samples were collected at 0 hr, 0.5hr, 1hr, 4hr, 9hr, and 24hr after chilling treatment for RNA preparation. In addition, after the chilling treatment, the plants were placed under normal growth condition from which leaf tissue samples were collected for RNA extraction at 24 hrs after returning the plants to the normal growing condition. The quantitative real-time PCR (qRT-PCR) showed that the induction of *SspNIP2* (~2.5 fold increase) was detectable as early as at 30 min after chilling treatment and maintained the induced level during the period of 24 hrs chilling stress condition (Fig 4).

### Phenotypic characteristics of *SspNIP2* transgenic tobacco plants

Since amino acid sequence and phylogenetic analyses suggested that SspNIP2 may function as a potential silicon transporter, and since it has been shown that silicon application improves crop tolerance to abiotic stresses (e.g. salt and drought stress) [20, 28], we investigated the potential function of *SspNIP2* under salt and drought stresses by generating transgenic tobacco plants expressing *SspNIP2* under dual 35S promoter. Through qRT-PCR, it was demonstrated that the expression level of *SspNIP2* greatly varied depending on transgenic lines, ranging from ~30 times to ~1,800 times increase compared to non-transgenic lines (S5 Fig). The transgenic lines showing a level of *SspNIP2* expression higher than 1,300 fold increase (high transgene expressor) developed chlorotic patches on the leaf margin of mature leaves (S5 Fig). These lines also expressed delayed flowering and lower number of viable seeds compared to control plants (data not shown). On the other hand, the transgenic lines showing less than 50 fold increase (low transgene expressor) showed no distinct phenotypic characteristics compared to the control group (S5 Fig).

### The effect of *SspNIP2* in transgenic tobacco plants under salt and water stress

**Fig 5:**
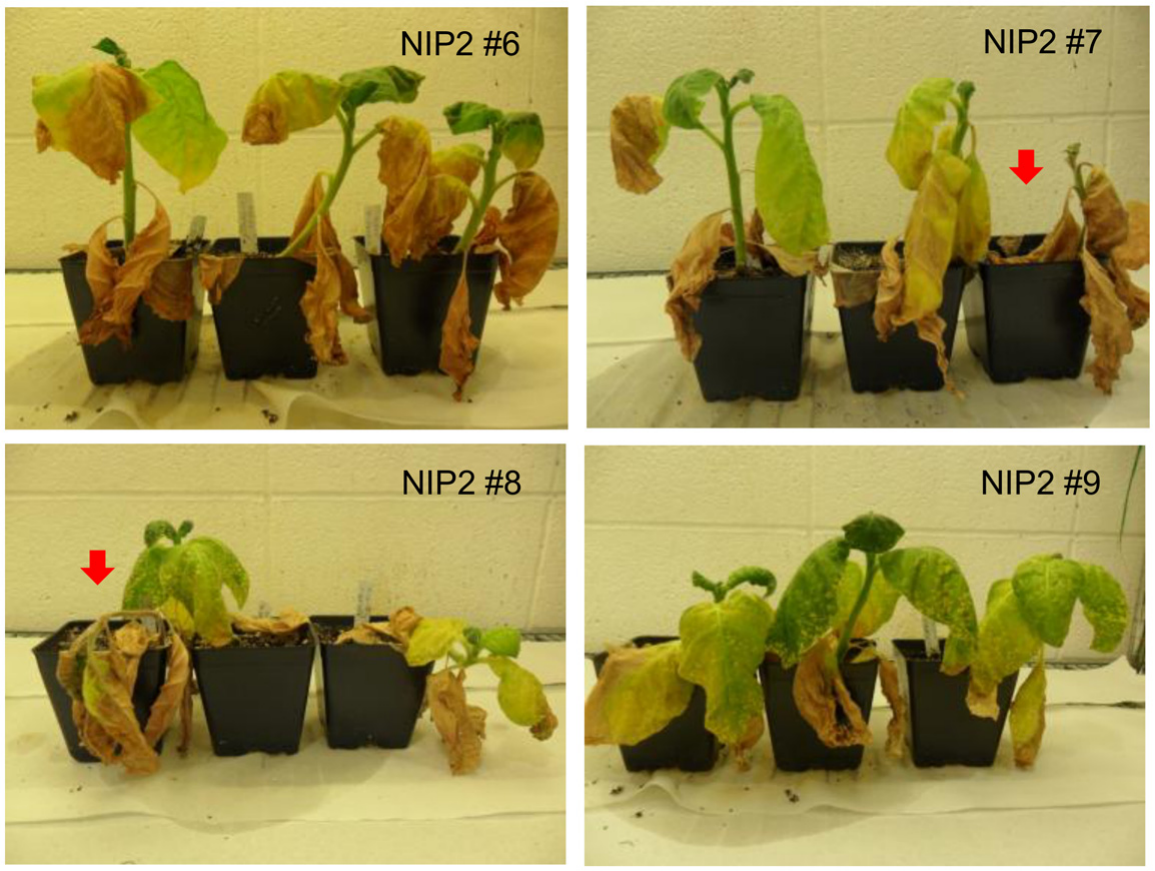
Salt stress test on SspNIP2 transgenic lines. Transgenic plants were irrigated with 100 ml of 200 mM NaCl for 4 weeks and 250 mM NaCl for another 2 weeks. Arrows indicate non-transgenic lines. NIP2 #6, #7, #8 and #9 referred to SspNIP2 transgenic line 6, 7, 8 and 9.

The SspNIP2 transgenic tobacco lines, NIP2 #6 and #7 (low transgene expresser; S5 Fig) and NIP2 #8 and #9 (high transgene expresser; S5 Fig), were subjected to high salt stress to investigate the potential function of SspNIP2 in plants under salinity stress condition. For salt stress test, six-week old transgenic and non-transgenic tobacco plants were irrigated once every three to four days with 100 ml of 200 mM NaCl for 4 weeks, followed by 100 ml of 250 mM NaCl for 2 weeks. Although all tested plants developed severe damage caused by high salt on the leaves, none of the wild type plants survived after 6 weeks of high salt treatment (Fig 5), suggesting some level of salt stress tolerance contributed by *SspNIP2* in the transgenic tobacco plants.

**Fig 6:**
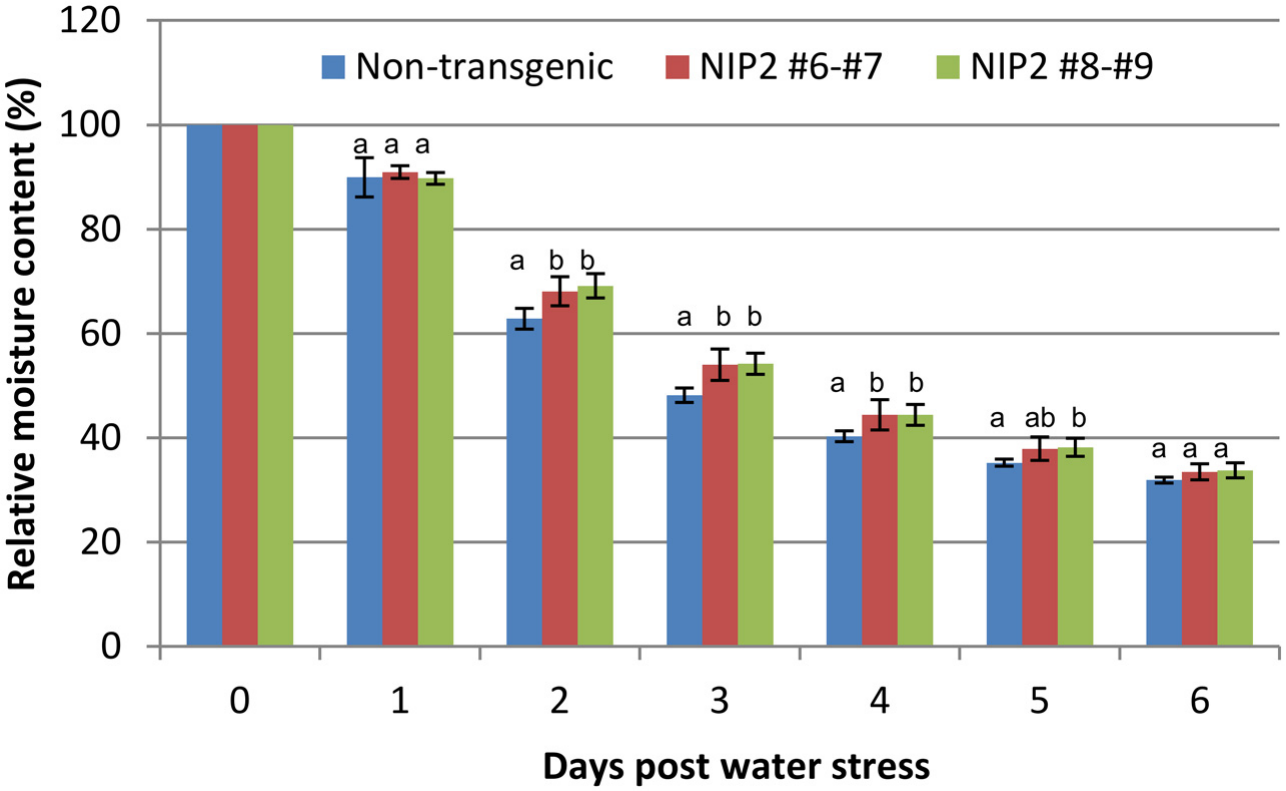
Water stress test on SspNIP2 transgenic lines. Relative moisture content (%) retained in each potted plant was measured during the first six days of water stress. The designation of transgenic lines are the same as shown in Fig 5. NIP2 #6-#7 and #8-#9 indicated that SspNIP2 transgenic lines #6 and #7, and lines #8 and #9 were combined, respectively, for the analysis. Statistical analysis was conducted by F-test followed by t-test.

In order to investigate the potential role of *SspNIP2* under water stress condition, six-week old transgenic lines of NIP2 #6, #7, #8 and #9, were subjected to water stress for 2 weeks without irrigation, and the weight of each pot was measured everyday to examine the moisture content retained in the pot of each plant during the period of experiment. After 2 weeks of water stress, all tested plants showed varying degrees of wilting from water stress (data not shown). Although a visual inspection failed to show a clear distinction between SspNIP2 transgenic and non-transgenic lines after 2 weeks of water stress, the measurement of relative moisture content clearly showed that SspNIP2 transgenic lines retained more moisture than the control group until 5 days post water stress, suggesting that the evapotranspiration rates of the SspNIP2 transgenic lines are lower than those of non-transgenic lines (Fig 6). The water stress data showed that the SspNIP2 transgenic lines showed a slight tolerance to early water stress condition as compared to the wild type plants.

## Discussion

Various abiotic stresses pose major constraints for maximum crop productivity in various climatic regions. It is known that cold-induced genes are also induced by water-stress, and vice-versa [29]. Furthermore, cross-talk among stress response genes, such as drought, high salinity and cold stress response have been reported [30, 31]. It is essential to understand the genetic mechanism of plant response towards abiotic stress, in order to better cope with various stress conditions imposed on crop species.

### Transcriptome analyses of a cold sensitive CP72-1210 and a cold tolerant TUS05-05 after chilling stress

RNA-Seq has been widely adopted for the study of gene expression profiles of various crop species under abiotic stress conditions [22, 32–35]. Dugas *et al*. [22] successfully analyzed the transcriptome of sorghum after water stress and hormone treatment, confirming the complex plant genetic networks against abiotic stress. As sequencing technology advances, the cost of sequencing decreased making RNA-Seq more affordable to many laboratories studying the so-called orphan crops. The current study utilized the efficiency of RNA-Seq on gene expression profiling of two *Saccharum* spp. genotypes, a cold susceptible sugarcane cultivar, CP72-1210, and a cold tolerant *S*. *spontaneum*, TUS05-05, in order to investigate *Saccharum* genes responding to cold stress. For this study, we utilized the sugarcane assembled sequences from SUCEST-FUN Database (http://sucest-fun.org) developed at Instituto de Química—Universidade de São Paulo, funded by FAPESP Bioenergy Research Program BIOEN (http://bioenfapesp.org), which hosted more than 43,000 expressed sequence tags (EST), the majority of which are functionally annotated. The RNA-Seq analysis with this database revealed that more than 600 SAS sequences are differentially expressed in each *Saccharum* genotype after cold stress. The functional annotation of those differentially expressed SAS sequences by Blast2Go showed that 51% and 47% of those differentially expressed SAS sequences in CP72-1210 and TUS05-05, respectively, were successfully annotated, which was less than expected. However, since SUCEST-FUN Database is the most comprehensive nucleotide sequence database currently available for sugarcane and other *Saccharum* species and is continuously updated by sugarcane research community, the annotation rate in the current study will be improved in near future when the new version of SUCEST-FUN Database is available.

The gene annotation revealed that the overall genetic response of CP72-1210 and TUS05-05 to cold stress were very similar. KEGG pathway mapping also showed that the major metabolic pathways (e.g. starch and sucrose metabolism, phenylpropanoid biosynthesis, purine and phenylalanine metabolism) that were affected by chilling stress were common in both genotypes. In fact, more than 76% of those differentially expressed genes in CP72-1210 and TUS05-05 was differentially regulated common in both genotypes. Despite the overall similarity in genetic responses against chilling stress in the cold sensitive CP72-1210 and the cold tolerant TUS05-05, the annotation analysis of those differentially regulated genes at the molecular function level revealed that the major difference in gene expression profiles between these two genotypes was on the genes involved in transmembrane transporter activities. The analysis of the genes that were up-regulated in genotype specific manner reconfirmed that the increased level of transmembrane transporter activity was observed only in the cold-tolerant TUS05-05. On the other hand, the cold sensitive CP72-1210 showed a stronger down-regulation of genes responding to various stresses (e.g. ethylene, salt and cold) than TUS05-05 as shown in S3 Fig and S1 Table. Further study is needed to see if those down-regulated genes involved in stress response are related to the sensitivity of chilling stress in CP72-1210.

Among those genes differentially expressed after cold stress, the expression of *SspNIP2*, a member of MIPs, was enhanced after cold stress in TUS05-05. MIPs are known to facilitate the transmembrane transport of water and other small uncharged polar molecules and can be grouped into four subfamilies: plasma intrinsic proteins (PIPs), tonoplast intrinsic proteins (TIPs), NIPs and small intrinsic proteins (SIPs) [24]. All MIPs except for SIPs, share a couple of common structural features, six transmembrane domains (H1 to H6) and two conserved NPA motifs in the loop B and E, respectively [24, 36, 37]. The ar/R region composed of four amino acid residues of MIPs is located in the narrowest region on the extra-membrane side of MIP pores and governs the substrate specificity of MIPs [24, 38–40]. Plant NIPs can be divided into three functional groups, NIP-I,-II, and-III, based on the four amino acid residues on the ar/R region of each NIP [27]. The ar/R selectivity filter of NIP-I is composed of WVAR four amino acid residues that is responsible for formamide, glycerol and moderate level of water transport [27]. The NIP-II functional group that is involved in urea transport has AIGR selective filter on the ar/R region with very low water permeability [27]. The third functional group of NIPs, NIP-III, functions as a silicon transporter that has GSGR residues on its ar/R selectivity filter [27]. The current study showed that the ar/R selectivity filter of SspNIP2 is composed of GSGR as in other monocot silicon transporters, suggesting that SspNIP2 may be involved in silicon transport in TUS05-05 of which expression is induced by cold stress. Preliminary data showed that the promoter region of *SspNIP2* harbors a cold responsive *cis*-acting DNA element (unpublished data). Phylogenetic analysis based on amino acid sequences of various monocot silicon transporter Lsi1s and Lsi6s [26, 41–44] indicated that SspNIP2 is grouped with Lsi6 silicon transporters which are involved in xylem unloading of silicon in the leaf tissue.

### Functional analysis of SspNIP2 in transgenic plants under salt and water stress

Quantitative RT-PCR showed that *SspNIP2* expression is induced at an early stage of cold stress indicating that the translocation of SspNIP2 substrate molecules takes place at the onset of the stress initiation. The salt and water stress test using SspNIP2 transgenic tobacco plants revealed that the SspNIP2 expression maintained the transgenic lines more vigorous than non-transgenic tobacco plants. As shown on amino acid sequence and phylogenetic analyses of SspNIP2 and other monocot silicon transporters, our data suggested that the improved stress tolerant phenotypes of SspNIP2 transgenic tobacco plants and TUS05-05 was mediated by SspNIP2 whose function may be similar to those of monocot silicon transporters. Currently investigations are underway to identify the substrate of SspNIP2 using transgenic tobacco and TUS05-05. Studies have shown that silicon application in crop species such as sugarcane, improves both biotic and abiotic stress tolerance leading to the increases in crop yield [28, 45–55]. Although it is not clear how silicon application contributes to biotic and abiotic stress tolerance in plants, previous studies have shown that the silicon accumulation in plant tissue provides a potential mechanical protection against drought stress and pathogen attack [49, 50] as well as a better regulated ion homeostasis under various abiotic stress conditions [51, 55–59].

In this report, we utilized SUCEST-FUN Database as a reference for *Saccharum* species RNA-Seq data analysis and demonstrated a streamlined procedure from transcriptome comparison between cold susceptible and tolerant genotypes of *Saccharum* species leading to the identification and validation of the functionality of one gene (named SspNIP2) on the improvement of abiotic stress tolerance in a heterologous plant system. The current study showed that, as the cost for next generation sequencing becomes more affordable, the RNA-Seq approach will provide a powerful tool to investigate the genes that contribute to abiotic stress tolerance in many economically important crop species with lack of reference genome.

## Supporting Information

S1 Fig Quantitative real-time PCR with six SAS sequences to validate the transcriptome data. The SAS sequence ID is indicated on the left of the graph where the fold increases of each SAS sequence in qRT-PCR and RNA-Seq were compared. The primer sequences are as follows: SCRLSD2009F04.g (forward: 5'-actgctgcttccttgtcttc-3', reverse: 5'-taacaccactcacgttcacg-3'); SCACLB1046H03.g (forward: 5'-caagtctgctgaggaggtg-3', reverse: 5'-gttttctgcctccttgagc-3'); SCCCCL3080G01.g (forward: 5'-gttttctggaccgattgctg-3', reverse: 5'-agaccgctgaggatgtgaag-3'); SCEPLB1042E05.g (forward: 5'-cgaacccacaaacacaatgg-3', reverse: 5'-aatgttgcgagggctaattg-3'); SCRFRZ3058E03.b (forward: 5'-aatccatccatccgtccaag-3', reverse: 5'-gccagccagacaacacctac-3'); SCUTSD1028A10.g (forward: 5'-aacgctgcaaaagaatatggag-3', reverse: 5'-ctggcctcaacacaacacct-3'). The primers for reference gene (GAPDH; glyceraldehyde-3-phosphate dehydrogenase) are 5'-aagggtggtgccaagaagg-3' (forward) and 5'-caaggggagcaaggcagtt-3' (reverse). The normalized value of gene expression level relative to the reference was calculated by 2^-ΔΔCt^.
(TIF)

S2 Fig Analysis of gene annotation of 237 differentially co-expressed genes commonly in CP72-1210 and TUS05-05. The annotated genes were analyzed based on the molecular function of GO terms by Blast2Go. GO terms are listed on the left, and the Blast2Go score of molecular function at level 3 is shown on top.
(TIF)

S3 Fig Analysis of gene annotation of differentially down-regulated genes unique in CP72-1210 (top) and TUS05-05 (bottom). The annotated genes were analyzed based on the biological process of GO terms by Blast2Go. GO terms are listed on the left, and the Blast2Go score of biological process at level 2 is shown on top of each graph.
(TIF)

S4 Fig Analysis of gene annotation of differentially up-regulated genes unique in CP72-1210 (top) and TUS05-05 (bottom). The annotated genes were analyzed based on the molecular function of GO terms by Blast2Go. GO terms are listed on the left, and the Blast2Go score of molecular function at level 2 is shown on top of each graph.
(TIF)

S5 Fig Transgene expression level and phenotypes of SspNIP2 transgenic tobacco plants. A. Quantification of SspNIP2 transgene expression level of SspNIP2 transgenic tobacco plants. NIP2 #6, #7, #8 and #9 referred to SspNIP2 transgenic line 6, 7, 8 and 9. The table showed the relative fold increase of SspNIP2 expression level in each transgenic line. The designation of low and high transgene expressing lines were based on the presence or absence of chlorotic patches on the leaves of each transgenic line. **B.** Comparison of leaf phenotypes of low and high transgene expressing lines.
(TIF)

S1 Table List of genes differentially expressed uniquely in TUS05-05 (Sheet: Unique in TUS05-05) and CP72-1210 (Sheet: Unique in CP72-1210), and commonly in both TUS05-05 and CP72-1210. (XLSX)

S2 Table A list of top four KEGG pathways obtained by KEGG pathway mapping with differentially expressed genes in CP72-1210 and TUS05-05 after chilling stress. (DOCX)
