# Supplementary figures and images for: Cold Responsive Gene Expression Profiling of Sugarcane and Saccharum spontaneum with Functional Analysis of a Cold Inducible Saccharum Homolog of NOD26-Like Intrinsic Protein to Salt and Water Stress
Source: PLoS One. 2015 May 4;10(5):e0125810. doi: 10.1371/journal.pone.0125810 (PMC4418668; doi:10.1371/journal.pone.0125810)

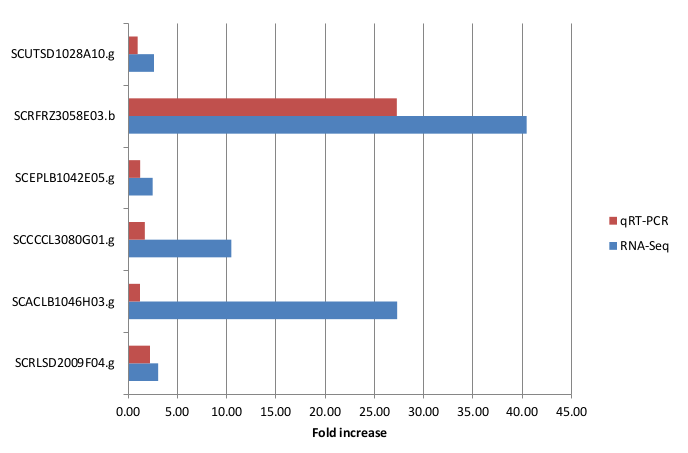

Supplement: S1 Fig — The SAS sequence ID is indicated on the left of the graph where the fold increases of each SAS sequence in qRT-PCR and RNA-Seq were compared. The primer sequences are as follows: SCRLSD2009F04.g (forward: 5'-actgctgcttccttgtcttc-3', reverse: 5'-taacaccactcacgttcacg-3'); SCACLB1046H03.g (forward: 5'-caagtctgctgaggaggtg-3', reverse: 5'-gttttctgcctccttgagc-3'); SCCCCL3080G01.g (forward: 5'-gttttctggaccgattgctg-3', reverse: 5'-agaccgctgaggatgtgaag-3'); SCEPLB1042E05.g (forward: 5'-cgaacccacaaacacaatgg-3', reverse: 5'-aatgttgcgagggctaattg-3'); SCRFRZ3058E03.b (forward: 5'-aatccatccatccgtccaag-3', reverse: 5'-gccagccagacaacacctac-3'); SCUTSD1028A10.g (forward: 5'-aacgctgcaaaagaatatggag-3', reverse: 5'-ctggcctcaacacaacacct-3'). The primers for reference gene (GAPDH; glyceraldehyde-3-phosphate dehydrogenase) are 5'-aagggtggtgccaagaagg-3' (forward) and 5'-caaggggagcaaggcagtt-3' (reverse). The normalized value of gene expression level relative to the reference was calculated by 2-ΔΔCt. (TIF) [file pone.0125810.s001.tif]

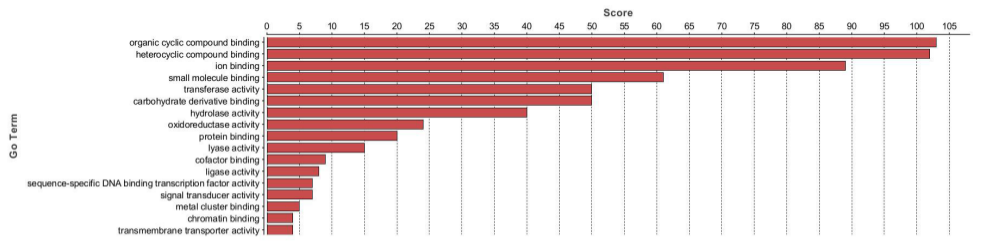

Supplement: S2 Fig — The annotated genes were analyzed based on the molecular function of GO terms by Blast2Go. GO terms are listed on the left, and the Blast2Go score of molecular function at level 3 is shown on top. (TIF) [file pone.0125810.s002.tif]

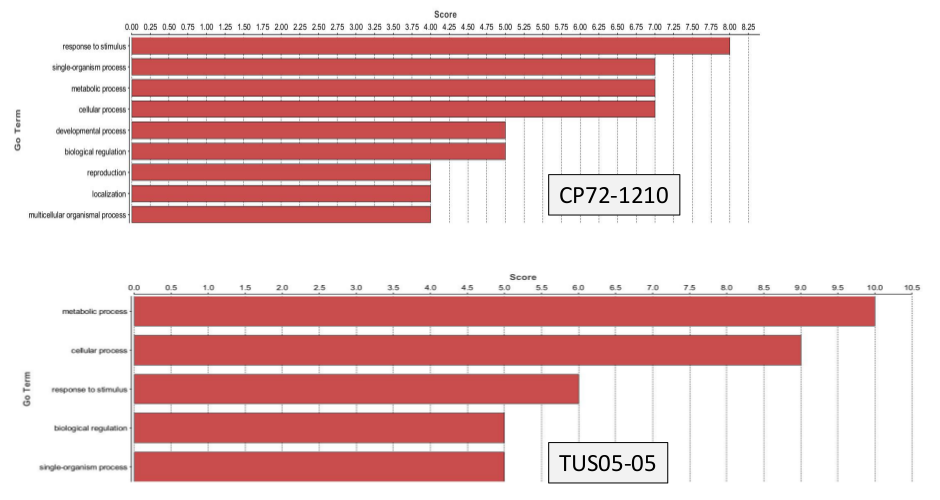

Supplement: S3 Fig — The annotated genes were analyzed based on the biological process of GO terms by Blast2Go. GO terms are listed on the left, and the Blast2Go score of biological process at level 2 is shown on top of each graph. (TIF) [file pone.0125810.s003.tif]

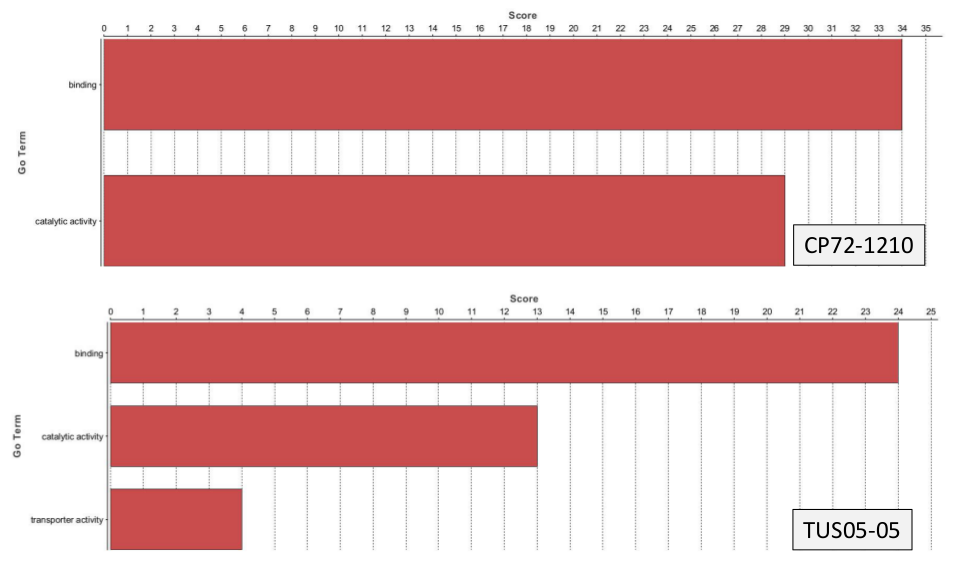

Supplement: S4 Fig — The annotated genes were analyzed based on the molecular function of GO terms by Blast2Go. GO terms are listed on the left, and the Blast2Go score of molecular function at level 2 is shown on top of each graph. (TIF) [file pone.0125810.s004.tif]

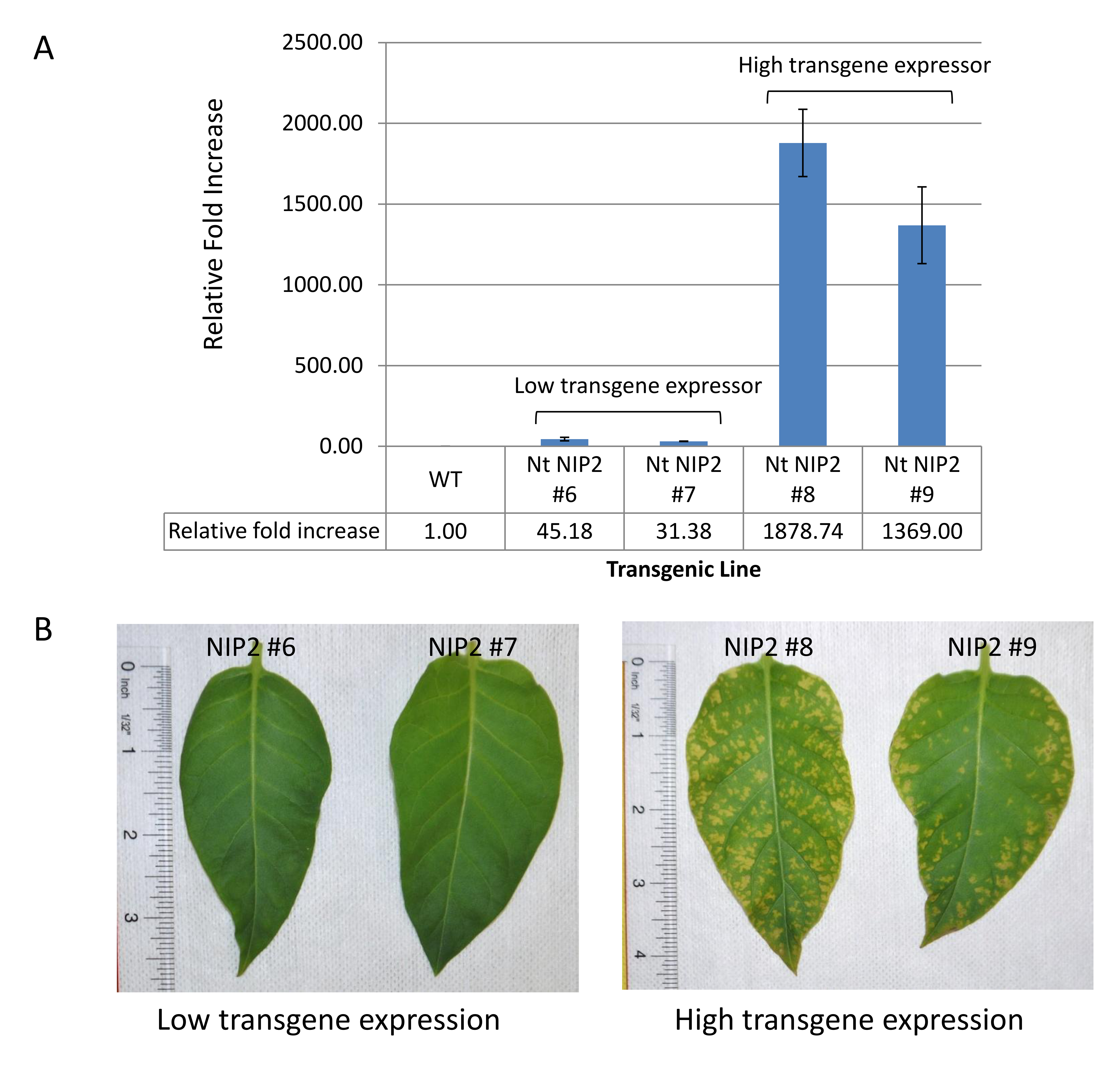

Supplement: S5 Fig — A. Quantification of SspNIP2 transgene expression level of SspNIP2 transgenic tobacco plants. NIP2 #6, #7, #8 and #9 referred to SspNIP2 transgenic line 6, 7, 8 and 9. The table showed the relative fold increase of SspNIP2 expression level in each transgenic line. The designation of low and high transgene expressing lines were based on the presence or absence of chlorotic patches on the leaves of each transgenic line. B. Comparison of leaf phenotypes of low and high transgene expressing lines. (TIF) [file pone.0125810.s005.tif]
