# Supplementary material for: Cold Responsive Gene Expression Profiling of Sugarcane and Saccharum spontaneum with Functional Analysis of a Cold Inducible Saccharum Homolog of NOD26-Like Intrinsic Protein to Salt and Water Stress
Source: PLoS One. 2015 May 4;10(5):e0125810. doi: 10.1371/journal.pone.0125810 (PMC4418668; doi:10.1371/journal.pone.0125810)
Supplement: S2 Table — (DOCX) [file pone.0125810.s007.docx]

**Table S2. A list of top four KEGG pathways obtained by KEGG pathway mapping with differentially expressed genes in CP72-1210 and TUS05-05 after chilling stress.**

| KEGG Pathway* | CP72-1210 | | TUS05-05 | |
| --- | --- | --- | --- | --- |
|  | Number of sequences | Number of enzymes | Number of sequences | Number of enzymes |
| Starch and sucrose metabolism | 14 | 7 | 14 | 6 |
| Phenylpropanoid biosynthesis | 12 | 5 | 8 | 3 |
| Purine metabolism | 11 | 6 | 13 | 6 |
| Phenylalanine metabolism | 10 | 4 | 7 | 2 |
